# A risk factor-based predictive model for new-onset hypertension during pregnancy in Chinese Han women

**DOI:** 10.1186/s12872-020-01428-x

**Published:** 2020-04-03

**Authors:** Yamin Hou, Lin Yun, Lihua Zhang, Jingru Lin, Rui Xu

**Affiliations:** 1 Department of Cardiology, Shandong Provincial Qianfoshan Hospital, Shandong University, Jinan, 250014 P.R. China; 2 Department of Cardiology, The First Affiliated Hospital of Shandong First Medical University, Jinan, 250014 P.R. China; 3 Department of Medicine, Jinan Maternity and Child Care Hospital, Jinan, 250001 P.R. China; 4 Department of Cardiology, Shandong Provincial Third Hospital, Jinan, 250031 P.R. China

**Keywords:** Hypertension, pregnancy induced, Prediction model, Risk factors, Homocysteine

## Abstract

**Background:**

Hypertensive disorders of pregnancy (HDP) is one of the leading causes of maternal and neonatal mortality, increasing the long-term incidence of cardiovascular diseases. Preeclampsia and gestational hypertension are the major components of HDP. The aim of our study is to establish a prediction model for pregnant women with new-onset hypertension during pregnancy (increased blood pressure after gestational age > 20 weeks), thus to guide the clinical prediction and treatment of de novo hypertension.

**Methods:**

A total of 117 pregnant women with de novo hypertension who were admitted to our hospital’s obstetrics department were selected as the case group and 199 healthy pregnant women were selected as the control group from January 2017 to June 2018. Maternal clinical parameters such as age, family history and the biomarkers such as homocysteine, cystatin C, uric acid, total bile acid and glomerular filtration rate were collected at a mean gestational age in 16 to 20 weeks. The prediction model was established by logistic regression.

**Results:**

Eleven indicators have statistically significant difference between two groups (*P* < 0.05). These 11 factors were substituted into the logistic regression equation and 7 independent predictors were obtained. The equation expressed including 7 factors. The calculated area under the curve was 0.884(95% confidence interval: 0.848–0.921), the sensitivity and specificity were 88.0 and 75.0%. A scoring system was established to classify pregnant women with scores ≤15.5 as low-risk pregnancy group and those with scores > 15.5 as high-risk pregnancy group.

**Conclusions:**

Our regression equation provides a feasible and reliable means of predicting de novo hypertension after pregnancy. Risk stratification of new-onset hypertension was performed to early treatment interventions in high-risk populations.

## Background

Hypertensive disorders of pregnancy (HDP) is a common placental-mediated syndrome complicated by various types of diseases during pregnancy and is also one of the leading causes of maternal and neonatal mortality. The disease manifests as hypertension, proteinuria and edema and is associated with serious complications such as hemolysis, elevated liver enzymes, and low platelet count (HELLP) syndrome, placental abruption, and stillbirth. Despite extensive clinical and basic research on HDP in recent decades, the real etiology and pathophysiology remain unclear [1, 2]. According to the latest recommendations from the International Society for the Study of Hypertension in Pregnancy (ISSHP), HDP includes pregnancy complicated by chronic hypertension (high blood pressure before pregnancy or increased blood pressure before a gestational age of 20 weeks) and new-onset hypertension [gestational hypertension (GH) or preeclampsia (PE)] [3]. PE and GH are the major components of HDP; their worldwide incidences are approximately 1.8–4.4% and 0.2–9.2%, respectively, and their incidences have regional and seasonal differences [4–6]. HDP increases the long-term incidence of cardiovascular diseases in pregnant women. Many studies have shown that the incidence of cardiovascular diseases in HDP patients is 2 times higher than that in normal pregnant women [7]. The occurrence of chronic hypertension in HDP patients is 1.5 times higher than that in normal pregnant women [8]. PE is an independent risk factor for the subsequent hypertension [9]. Basal blood pressure and the long-term incidence of cardiovascular diseases are higher in children born by pregnant women with hypertension [10]. The personal and social burden caused by HDP is very serious. Therefore, studies on the risk factors of HDP have always been on-going.

Currently, the most studied risk factors include maternal age, multiple pregnancies, obesity, and family history of PE, previous PE, chronic hypertension, and genetics. However, the specificity and sensitivity of these risk factors remain questionable [11–14]. There are many risk factors for HDP, and none of them have been used as a gold standard for HDP screening. A study demonstrated that none of the classical risk factors alone reached an acceptable discriminatory accuracy [15]. The establishments of risk factor models have mostly focused on predicting the risk of PE and have not concentrated on GH. However, the ISSHP believes that the incidence of GH is constantly increasing and that GH will convert to PE in some patients in the late stage, and it classifies HDP as chronic hypertension and de novo hypertension during pregnancy (GH and PE). The ISSHP also questions the existing diagnostic criteria for PE, suggesting that proteinuria cannot be used as a mandatory criterion for the diagnosis of PE and that if liver and kidney dysfunction are present, PE needs to be considered. Therefore, the purpose of this study was to combine the clinical and biochemical indices of patients to establish a risk model for new-onset hypertension (GH and PE) in Chinese Han women and to provide a theoretical basis for the prediction and prevention of HDP progression.

## Methods

### Enrolled populations

Participants who underwent physical examination and gave birth in hospital were recruited from January 2017 to June 2018. Case control study design was adopted. Patients with hypertension during pregnancy were selected as case group (gestational hypertension, preeclampsia) and normal pregnancy as control group.

### Case group and control group inclusive and exclusive criteria

The case group inclusion criteria were as follows: age over 18, women who had a singleton pregnancy, received antenatal care after 16 to 20 weeks and delivered in the same maternity unit were eligible for inclusion, and diagnosed in gestational hypertension or preeclampsia after discharge and the control group diagnosed no hypertension. The exclusion criteria were as follows: (1) secondary hypertension (primary aldosteronism, pheochromocytoma, Cushing syndrome, etc.), (2) patients with chronic hypertension combined with pregnancy and superimposed preeclampsia, (3) severe heart failure and liver and kidney failure, (4) chronic kidney disease and renal vascular disease, (5) acute and severe diseases in obstetrics and gynecology (amniotic fluid embolism, eclampsia, HELLP syndrome, etc.), (6) tumor, (7) new serious infections, (8) severe anemia (hemoglobin < 60 g/L), (9) multiple organ failure.

### Data collection

Maternal clinical parameters were included age, height, weight, family history of HDP. Maternal fasting blood samples were collected after an overnight fast of at least 8 h at a mean gestational age of 16 to 20 weeks. The biomarkers were included white blood cell count (WBC), red blood cell count (RBC), hemoglobin (HB) and platelets (PLT), albumin (ALB), globulin (GLB), glucose (Glu), free fatty acids (FFA), direct bilirubin (DBil), indirect bilirubin (IBil), homocysteine (HCY), cystatin C (CYC), uric acid (UA), total bile acid (TBA), serum calcium (Ca), serum zinc (Zn), estimated glomerular filtration rate (eGFR).

### Data analysis

A total of 316 patients were enrolled: 199 healthy pregnant women and 117 pregnant women with GH or PE. The body mass index (BMI) of each sample was calculated using patient height and weight. All data were analysed using SPSS23.0 (SPSS Inc., Chicago, IL, USA). Measurement data are expressed as the means± standard deviations (x ± s), except for the HCY, TBA, Dbil, IBil, FFA (M, P_25_-P_75_). Counting data are expressed as proportions (%). An independent-samples t-test was performed for age, CYC, ALB, GLB, Glu, UA, Ca, Zn, BMI, eGFR, WBC, RBC, Hb, and PLT, and Mann-Whitney U tests was performed for HCY, TBA, DBil, IBil, FFA. Meaningful factors were converted to ordinal variables based on percentiles, except the age for five groups cause the age range is small. The obtained ordinal variables and family history were subjected to the Chi-square test to determine the *P* value. Meaningful factors were subjected to collinearity diagnosis to clarify the presence of collinear interactions among them. After excluding the influence of collinearity, each factor was substituted into a binary logistic regression equation to screen out meaningful independent risk factors and establish a risk factor-based prediction model for pregnancy induced hypertension. The area under the ROC curve (AUC), the optimal cutoff point and the 95% confidence interval (CI) of the model were calculated to evaluate its validity. The scores were calculated according to the regression coefficient β of each variable in the logistic regression prediction model, i.e., the score of each variable was the integer value of the absolute value of each variable β divided by the minimum value of the absolute value of each variable β. The scores for each study subject were calculated to establish a scoring system. The AUC was used for evaluation.

## Results

The difference between the two groups was statistically significant in age (29.410 ± 4.803, 31.460 ± 5.253, *P*<0.001), HCY[10.600(6.600–14.800), 14.000(10.100,18.900), *P*<0.001)], CYC (1.081 ± 0.289, 1.376 ± 0.339, *P*<0.001), UA (275.820 ± 72.209, 350.040 ± 94.962, *P*<0.001), TBA[2.100(1.400–3.200), 3.400(1.500–5.100), *P*<0.001], BMI (28.457 ± 3.348, 30.766 ± 3.793, *P*<0.001), calcium (2.148 ± 0.103, 2.081 ± 0.223, *P* = 0.003), zinc (8.248 ± 1.599, 7.727 ± 1.972, *P* = 0.016), ALB (33.360 ± 4.136, 29.997 ± 3.911, *P*<0.001), eGFR (222.933 ± 66.143, 187.506 ± 58.359, *P*<0.001). However, there was no statistically significant group difference for GLB, FFA, DBil, IBil, Glu, WBC, RBC, PLT and Hb as shown in Table 1.

**Table 1:** Comparison of basic and clinical indicators between the 2 groups

Item	Control group (***n*** = 199)	Case group (***n*** = 117)	***P*** value
**Age (yr)**	29.410 ± 4.803	31.460 ± 5.253	<0.001
**HCY (μmol/L)**	10.600(6.600–14.800)	14.000(10.100,18.900)	<0.001
**Height (cm)**	161.580 ± 4.855	161.210 ± 4.434	0.497
**Weight (kg)**	74.299 ± 9.257	79.914 ± 10.076	<0.001
**UA (μmol/L)**	275.820 ± 72.209	350.040 ± 94.962	<0.001
**CYC (mg/L)**	1.081 ± 0.289	1.376 ± 0.339	<0.001
**TBA (μmol/L)**	2.100(1.400–3.200)	3.400(1.500–5.100)	<0.001
**Calcium (mmol/L)**	2.148 ± 0.103	2.081 ± 0.223	0.003
**BMI (kg/m**^**2**^**)**	28.457 ± 3.348	30.766 ± 3.793	<0.001
**Zinc (μmol/L)**	8.248 ± 1.599	7.727 ± 1.972	0.016
**Albumin (g/L)**	33.360 ± 4.136	29.997 ± 3.911	<0.001
**Globulin (g/L)**	25.175 ± 3.618	24.553 ± 3.275	0.128
**WBC (10**^**9**^**/L)**	10.068 ± 2.487	10.698 ± 3.174	0.069
**RBC (10**^**9**^**/L)**	3.743 ± 0.411	3.756 ± 0.565	0.796
**HB(g/L)**	105.620 ± 14.201	109.220 ± 17.550	0.062
**PLT (10**^**9**^**/L)**	221.810 ± 61.384	218.670 ± 69.487	0.678
**DBil (μmol/L)**	2.400(1.900–3.200)	2.400(1.900–3.050)	0.562
**IBil (μmol/L)**	2.750(1.770–4.000)	2.200(1.400–3.900)	0.090
**FFA (mmol/L)**	0.460(0.340–0.610)	0.490(0.350–0.640)	0.594
**Glu (mmol/L)**	4.740 ± 1.408	5.050 ± 1.292	0.061
**FH (yes/no)**	64.8%/28.6%	35.2%/71.4%	0.090
**EGFR (ml/min/1.73 m2)**	222.933 ± 66.143	187.506 ± 58.359	<0.001

*HCY* Homocysteine, *UA* Uric acid, *CYC* Cysteine, *TBA* Total bile acid, *BMI* Body mass index, *EGFR* Estimated glomerular filtration rate, *WBC* White blood cell count, *RBC* Red blood cell count, *HB* Hemoglobin, *PLT* Platelets, *DBil* Direct bilirubin, *IBil* Indirect bilirubin, *FFA* Free fatty acids, *Glu* Glucose, *FH* Family history

The above continuous variables with significant differences were converted into grade variables, shown in Table 2. According to the Chi-square, the *P* values of age, HCY, CYC, UA, TBA, BMI, eGFR, ALB, calcium, zinc and family history of hypertensive disorders were less than 0.05, showed statistically significant differences.

**Table 2:** Ordinary variables

Item	0	1	2	3	4
**Age (yr)**	≤26.000	(26.000,29.000]	(29.000,31.000]	(31.000,34.000]	>34.000
**HCY (μmol/L)**	≤7.930	(7.930,12.000]	(12.000,16.180]	>16.180	
**UA (μmol/L)**	≤236.000	(236.000,294.000]	(294.000,352.000]	>352.000	
**CYC (mg/L)**	≤0.970	(0.970,1.130]	(1.130,1.350]	>1.350	
**Albumin (g/L)**	≤29.830	(29.830,32.600]	(32.600,34.180]	>34.180	
**TBA (μmol/L)**	≤1.500	(1.500,2.400]	(2.400,4.000]	>4.000	
**Ca (mmol/L)**	≤2.060	(2.060,2.140]	(2.140,2.210]	>2.210	
**Zinc (μmol/L)**	≤7.000	(7.000,8.000]	(8.000,8.900]	>8.900	
**BMI (kg/m**^**2**^**)**	≤26.350	(26.350,29.320]	(29.320,31.820]	>31.820	
**EGFR (ml/min/1.73 m2)**	≤158.630	(158.630,203.830]	(203.830,254.130]	>254.130	

*CYC* Cystatin C, *UA* Uric acid, *HCY* Homocysteine, *EGFR* Estimated glomerular filtration rate, *BMI* Body mass index, *TBA* Total bile acid, *Ca* Calcium

The 11 factors do not exist multicollinearity, the binary logistic model build by 11 variables is stable. The binary logistic regression showed ALB (0.489, 95% CI: 0.335–0.714) was the protective factor. HCY (1.400, 95% CI: 1.051–1.864), CYC (1.795, 95% CI: 1.272–2.534), UA (1.667, 95% CI: 1.205–2.306), BMI (1.589, 95%CI: 1.161–2.175), age (1.289, 95% CI: 1.026–1.620), and TBA (1.721, 95% CI: 1.277–2.320) were risk factors, showed on Table 3.

**Table 3:** Binary logistic regression

Item	β	SE	P	OR	95%CI lower	95%CI upper
**CYC**	0.585	0.176	0.001	1.795	1.272	2.534
**Albumin**	−0.715	0.193	<0.001	0.489	0.335	0.714
**UA**	0.511	0.166	0.002	1.667	1.205	2.306
**Calcium**	−0.027	0.181	0.879	0.973	0.682	1.387
**Zinc**	−0.107	0.149	0.471	0.898	0.671	1.203
**EGFR**	−0.240	0.169	0.156	0.787	0.565	1.096
**BMI**	0.463	0.160	0.004	1.589	1.161	2.175
**Age**	0.254	0.116	0.029	1.289	1.026	1.620
**HCY**	0.336	0.146	0.021	1.400	1.051	1.864
**TBA**	0.543	0.152	<0.001	1.721	1.277	2.320
**FH**	0.918	0.799	0.251	2.503	0.523	11.988
**Constant**	−4.982	1.088	0.000	0.007		

*CYC* Cystatin C, *UA* Uric acid, *HCY* Homocysteine, *EGFR* Estimated glomerular filtration rate, *BMI* Body mass index, *TBA* Total bile acid, *FH* Family history

**Fig. 1:**
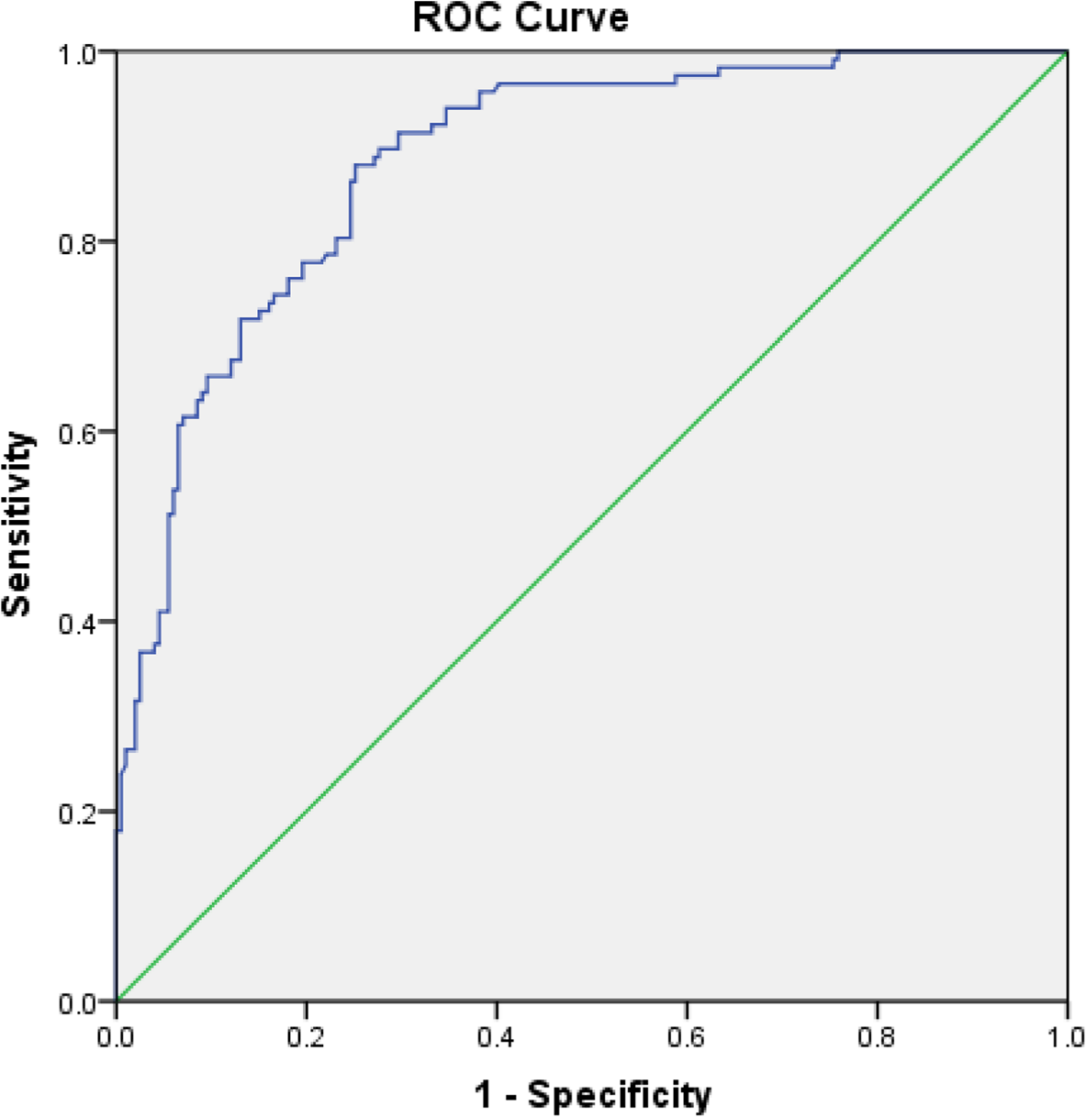
ROC curve of binary logistic regression. The AUC was 0.884(95%CI: 0.848–0.921). The sensitivity is 88.0%, and the specificity is 75.0%

**Fig. 2:**
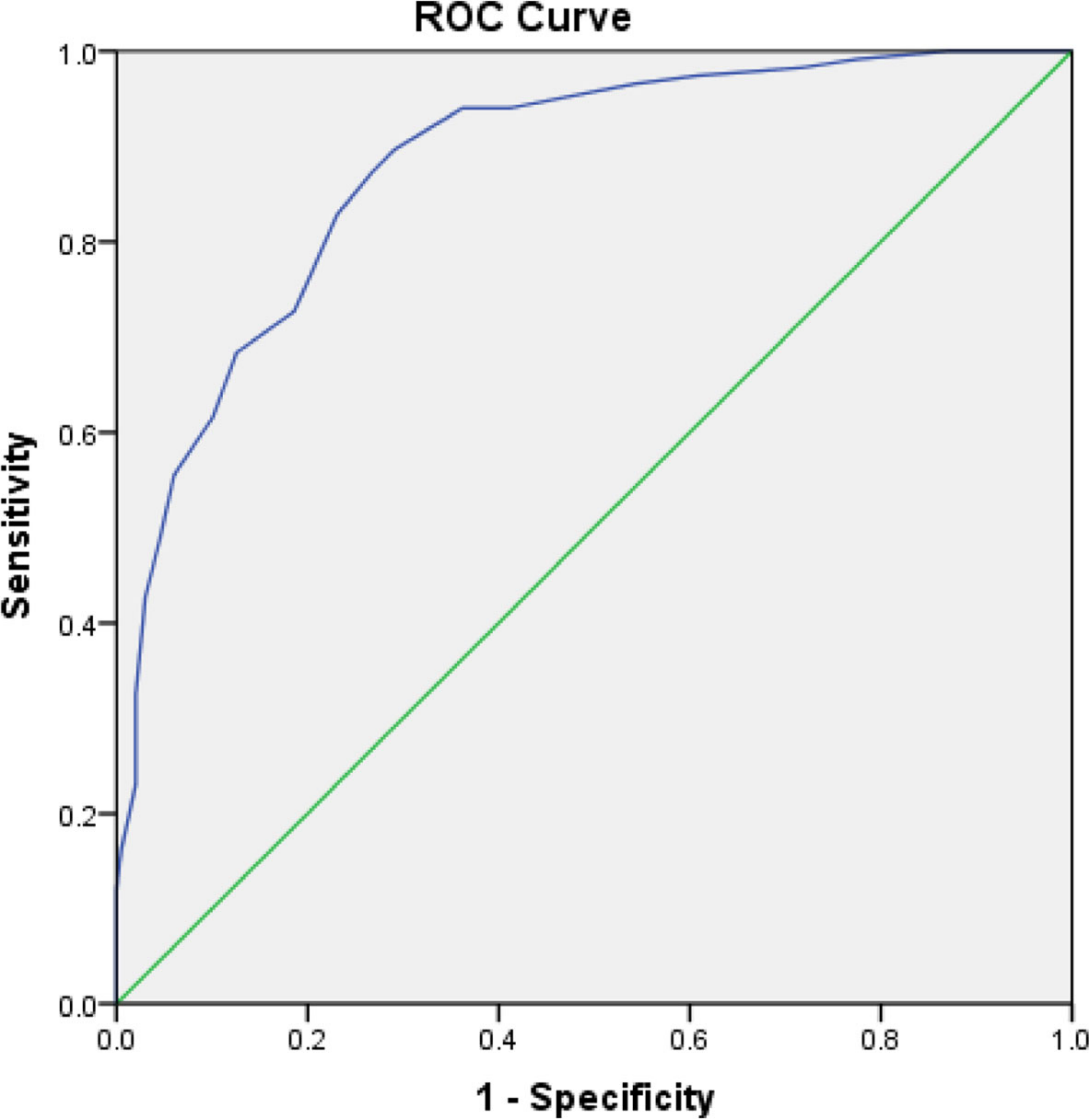
ROC curve of scoring system. The AUC was 0.880(95%CI 0.842–0.918). The sensitivity is 89.7%, the specificity is 70.9%

In order to optimize the model, logistic regression was performed again for 7 independent predictors, and the equation was: Y = (− 5.855) + 0.590 (CYC) - 0.766 (ALB) + 0.591 (UA) + 0.399 (BMI) + 0.305 (age) + 0.332 (HCY) + 0.526 (TBA), as shown in Table 4. The AUC was 0.884(95%CI: 0.848–0.921). The maximum value of the Youden index is 0.629, the sensitivity is 88.0%, and the specificity is 75.0%, as shown in Fig. 1. The scoring system was established according to the optimized model, and the scores of each factor were obtained, as shown in Table 5. ROC curve was drawn for the scoring system, with the AUC being 0.880(95%CI 0.842–0.918). The maximum value of its Youden index is 0.606, the sensitivity is 89.7%, the specificity is 70.9%, and the corresponding cut-off value is 15.5, as shown in Fig. 2.

**Table 4:** Optimal binary logistic regression

Item	β	SE	P	OR	95%CI lower	95%CI upper
**CYC**	0.590	0.165	<0.001	1.805	1.307	2.492
**Albumin**	−0.766	0.155	<0.001	0.465	0.343	0.630
**UA**	0.591	0.156	<0.001	1.805	1.330	2.449
**BMI**	0.399	0.146	0.006	1.490	1.120	1.983
**Age**	0.305	0.109	0.005	1.356	1.096	1.678
**HCY**	0.332	0.142	0.020	1.393	1.054	1.842
**TBA**	0.526	0.147	<0.001	1.692	1.269	2.256
**Constant**	−5.855	0.964	<0.001	0.003		

*CYC* Cystatin C, *UA* Uric acid, *BMI* Body mass index, *HCY* Homocysteine, *TBA* Total bile acid

**Table 5:** Scoring system

Variance	Score
**CYC**	
**≤ 0.970**	0
**(0.970,1.130]**	2
**(1.130,1.350]**	4
**>1.350**	6
**Age**	
**≤ 26.000**	0
**(26.000,29..000]**	1
**(29.000,31.000]**	2
**(31.000,34.000]**	3
**>34.000**	4
**HCY**	
**≤ 7.930**	0
**(7.930,12.000]**	1
**(12.000,16.180]**	2
**>16.180**	3
**UA**	
**≤ 236.000**	0
**(236.000,294.000]**	2
**(294.000,352.000]**	4
**>352.000**	6
**Albumin**	
**≤ 29.830**	0
**(29.830,32.600]**	−2
**(32.600,34.180]**	−4
**>34.180**	−6
**BMI**	
**≤ 26.350**	0
**(26.350,29.320]**	1
**(29.320,31.820]**	2
**>31.820**	3
**TBA**	
**≤ 1.500**	0
**(1.500,2.400]**	2
**(2.400,4.000]**	4
**>4.000**	6

*CYC*Cystatin C, *UA* Uric acid, *BMI* Body mass index, *HCY* Homocysteine, *TBA* Total bile acid

## Discussion

We collected 199 healthy pregnant women, 117 pregnant women with GH or PE and 22 variables. 11 indicators have significance and 7 indicators are independent risk factors. HCY is a special risk factor for hypertension to the Chinese Han population. Our results showed that HCY was closely associated with the incidence of new-onset HDP in Chinese Han pregnant women [odds ratio (OR): 1.393, 95% CI: 1.054 to 1.842] in the optimal model. HDP is an idiopathic disease with a complex mechanism that has a tremendous impact on the health of women and their children during pregnancy and the postpartum period. Studies are continuously being carried out on the risk factors that affect HDP development, and the guidelines are constantly changing. Therefore, risk factor-based models have been constantly designed and developed. Goetzinger and Verlohren et al. established a risk model for pregnant women based on high-risk factors and serum biochemical indicators such as pregnancy-associated plasma A (PAPP-A) and placenta growth factor (PIGF), but the receiver operating characteristic (ROC) curve was poor [16, 17]. The 2018 ISSHP guidelines classify HDP into 2 categories: chronic hypertension and new-onset hypertension (GH or PE) that occurred 20 weeks after pregnancy [3]. Therefore, in our study, GH and PE were classified as new-onset hypertension during pregnancy and were included in the case group, and normal pregnant women were used as the control group for the case-control study to establish a risk factor-based model for new-onset HDP in the Chinese Han population.

The multivariable analysis results in this study showed that albumin, CYC, UA, HCY, BMI, age, and TBA were independent predictive factors of HDP. Hyperhomocysteinemia is a specific disease in the Chinese Han population. The TT genotype caused by the C-T mutation in the C677T gene polymorphism of the key metabolic enzyme MTHFR hinders the conversion of HCY to methionine and thus causes hyperhomocysteinemia. The population with the TT genotype accounts for 25% of the Chinese Han population, whereas the population with the TT genotype in North America and Europe accounts for only approximately 12% of the combined population [18]. Hyperhomocysteinemia causes vascular endothelial inflammation, which causes changes in vascular endothelial cells and the proliferation of smooth muscle cells, leading to vascular diseases [19]. Our study found that the risk of new-onset hypertension increases by nearly 1.4-fold with a quartile increase in HCY compared to control group. PE is a systemic endothelial dysfunction. HCY is likely related to the pathogenesis of HDP by vascular-mediated oxidative stress or direct injury [20]. Hyperhomocysteinemia is defined as a plasma HCY level greater than 10 μmol/L [21]. Our data showed that the rate of hyperhomocysteinemia in normal pregnant women was 55.8% and that the rate of hyperhomocysteinemia in patients with HDP was 76.1%. This difference was statistically significant. Therefore, our study suggested that an increase in HCY increases the risk of HDP and that the population with HDP is more susceptible to hyperhomocysteinemia. The other study has demonstrated that the concentrations of HCY are associated with PE and the concentration of HCY in the first trimester was an independent risk factor for the development of severe PE [22]. A Large meta-analysis also indicated that the average HCY level was significantly higher in PE women than the normal pregnant women [23].

This study found that uric acid was also a risk factor for the development of HDP and that the risk of HDP increased by 1.805-fold for each quartile increase in uric acid. The finding is consistent with that of Nair et al. [24] and Wu et al. [25]. Some studies suggest that the increase in uric acid can reduce the production of nitric oxide in endothelial cells, leading to reduced trophoblast invasion and resulting in a lack of physiological transformation of the spiral artery, thus causing HDP, and that a deficiency in nitric oxide can lead to reduced vascular tension, resulting in PE [26]. Weissgerber and Brown believe that hyperuricemia leads to maternal endothelial dysfunction and affects placental blood flow, suggesting that uric acid is the precursor of PE [27, 28]. This is consistent with our findings. CYC is a relatively stable indicator that is not easily affected by nonrenal factors and can remain relatively stable throughout gestation [29]. Our study showed that CYC was positively correlated with the occurrence of HDP, which is consistent with the study by Novakov Mikic et al. [30].

Our study also showed that BMI was positively correlated with the occurrence of HDP and was an independent risk factor for the prediction of HDP, which was consistent with the studies by Marchi J [31] and Salihu [32]. Age is still a controversial clinical predictor of HDP. Some studies have shown that advanced age or young age is associated with a higher risk of HDP [33], while Paré et al. found that age greater than 40 years was significantly correlated with HDP [34]. There have also been many studies that suggest no significant correlation between age and the onset of HDP [35, 36]. Our study showed that age was positively correlated with the risk of HDP; the relative risk was 1.356.

The pathophysiological basis of the development of HDP is vascular endothelial cell damage, which increases vascular permeability and can cause hypoalbuminemia and edema. Therefore, Dai et al. proposed that serum albumin is a protective factor for PE [37]. Our study showed that albumin was negatively correlated with the incidence of HDP; that is, the higher the serum albumin concentration was, the lower the risk of HDP. Albumin leakage is not limited to the interstitial space. Due to glomerular endothelial cell damage, the infiltration of albumin increases in PE. This symptom is not limited to PE. The combined analysis of GH and PE showed that albumin was correlated with the risk of pregnancy induced hypertension; the risk was 0.489. Even in the absence of proteinuria or edema in pregnant women with hypertension, decreased serum albumin concentration should be a cause of concern.

Total bile acid is an indicator that reflects intrahepatic cholestasis of pregnancy (ICP); ICP was once thought to be unrelated to liver diseases caused by PE; however, with progress in research, it was found that ICP may cause PE [38]. The diagnosis of ICP is complicated, and its evaluation indicators include pruritus and elevated liver enzymes. Total bile acid was the index that was focused on in this study, and it was found to be significantly positively correlated with new-onset hypertension. The prevention and treatment of HDP relies on monitoring total bile acid.

The predictive model of risk factors for HDP provided in this study was used to analyze new-onset hypertension (GH and PE) as the case group. In the past 10 years, studies on HDP focused on PE risk prediction of but ignored GH. This was a case-control study comparing the risk factor-based models of HDP reported in case-control studies in the past 15 years. Direkvand-Moghadam et al. [39] established a prediction model using a medical history of PE, hypertension and infertility. The AUC of the model was 0.67 (95% CI: 0.59–0.67, *p*< 0.01). The specific parameters, such as PIGF, free b-human chorionic gonadotropin (β-hCG), PAPP-A and placental protein-13 (PP-13), which were not widely studied in clinical practice, were included in the study, and the model had a sensitivity of 70% [40]. Another study constructed the model that include the instructors reflective of placental function (PAPP-A and PlGF) and of the resistance in the uteroplacental circulation (uterine artery pulsatility index) could identify more than 90% of cases of early PE [41]. A large-scale systematic review summarized 29 risk prediction models for HDP, in which most of them predicted the risk of PE; the AUC of the prediction efficiency of the 29 models was between 0.67 and 0.90 [42]. Since then, most of the risk predictive model are included the maternal parameters, the instructors reflective of placental function such as PAPP-A, β-hCG or the PIGF. Little of the models covered the HCY or the kidney and liver function. The indicators included in our prediction model are more comprehensive, including maternal characteristics, family history, and metabolic indicators of the kidney and liver. We incorporated HCY and a series of biochemical indicators of liver and kidney function into our model innovatively. These indicators are easy to apply clinically and are cost-effective, and the AUC, sensitivity and specificity of our prediction model was 0.884 (95% CI: 0.848–0.921), 88.0%, and 75.0%, respectively. Compared to the aforementioned models, this model has high quality in terms of efficacy, and its sensitivity and specificity were excellent. As for other factors we have collected such as family history of HDP, in other study was proved to be the risk factor for HDP. Family history of HDP has been studied for a long time; a Norwegian cohort study now showed that pregnant women with a family history of HDP are more likely to develop hypertension during pregnancy, which is thought to be associated with family genetic phenotypes [43]. The genetic variants also have effect on HDP, among them; the M235T variant of the angiotensinogen gene has been shown in many subjects. In a Japanese study, the M235T frequency of the TT genotype is significantly higher in primigravid HDP (93–94%) than controls (77%) [44]. A meta-analysis suggested that MTHFR C677T polymorphism was associated with preeclampsia in Asian and Caucasian populations [14]. Because of genetic variants is not easy to carry out in clinical, we did not include it in the study. Other factors such as the intake of calcium have been proved to be the risk factors of HDP. According to a meta-analysis, the additional intake of calcium during pregnancy is an effective measure to reduce the incidence of preeclampsia, especially in populations at high risk [45]. Women who are primiparous also show a higher risk of HDP [2]. Some placenta-related factors may also predict the risk of HDP especially the PE. Increased production of soluble fms-like tyrosine kinase receptor-1 (sFlt-1) and soluble endoglin (sEng), and decreased circulating levels of free vascular endothelial growth factor (VEGF), contribute to the pathophysiology of PE. Cheng has studied the sFlt-1, sEng and VEGF levels showed a significant correlation with in the PE groups but not in the normotensive group [46]. Although the placenta-related factor has been studied widely in recent years, it is still difficult to carry out in clinical, so we did not include these factors in the study. The limitation of this prediction model is the lack of an external validation model. We are constantly collecting data to establish external validation models, and we will further improve the model according to the subsequent results to better serve in the clinical.

For clinical application, the risk factor-based prediction model for HDP in this study was Y = (− 5.855) + 0.590 (CYC) -0.766 (ALB) + 0.591 (UA) + 0.399 (BMI) + 0.305 (age) + 0.332 (HCY) + 0.526 (TBA). The AUC of this model is 0.884. For clinical, if we evaluated these factors together in the early gestational weeks, the predictive efficiency for later HDP was 88.4%. We established a scoring system to simplify the risk evaluation. The AUC, sensitivity, specificity and cutoff value were 0.880, 89.7%, 70.9%, and 15.5, respectively. The predictive efficiency of this scoring system is also more than 80%. We tested the above 7 indicators for pregnant women, and the results were evaluated by the scoring system, those whose total score exceeded 15.5 were high-risk groups, while those whose total score was less than 15.5 were low-risk groups. In clinical, the 7 indicators are recommended for the screening of HDP, score can be made according to the indicators of pregnant women, and the risk stratification can be made according to the score. This predictive model has provided certain help to the judgment of doctors for early screening of high-risk groups and focused surveillance for this population.

## Conclusion

Age, HCY, CYC, uric acid, total bile acid, BMI, and albumin were independent predictors of gestational hypertension and preeclampsia. The risk factor prediction model and scoring system established by these 7 items have high sensitivity and specificity, which can provide good estimates to the risk stratification of de novo hypertension in pregnancy.

## Data Availability

Study protocol and data set: Not available. Statistical code: Available from Dr. Xu (e-mail, xuruicn@hotmail.com).

## Abbreviations

HDP: Hypertensive disorders of pregnancy
HELLP syndrome: Hemolysis, elevated liver enzymes, low platelet syndrome
ISSHP: International Society for the Study of hypertension in pregnancy
GH: Gestational hypertension
PE: Preeclampsia
PAPP-A: Pregnancy-associated plasma A
PIGF: Placenta growth factor
ROC: Receiver operating characteristic
WBC: White blood cell count
RBC: Red blood cell count
HB: Haemoglobin
PLT: Platelets
ALB: Albumin
GLB: Globulin
Glu: Glucose
FFA: Free fatty acids
DBil: Direct bilirubin
IBil: Indirect bilirubin
HCY: Homocysteine
CYC: Cystatin C
UA: Uric acid
TBA: Total bile acid
Ca: Serum calcium
Zn: Serum zinc
eGFR: Estimated glomerular filtration rate
AUC: Area under the receiver operating characteristic curve
CI: Confidence interval
ICP: Intrahepatic cholestasis of pregnancy
β-hCG: β-human chorionic gonadotropin
PP-13: Placental protein-13
sFlt-1: Fms-like tyrosine kinase receptor-1
sEng: Soluble endoglin
VEGF: Vascular endothelial growth factor
